# From Single Nucleotide Polymorphisms to Constant Immunosuppression: Mesenchymal Stem Cell Therapy for Autoimmune Diseases

**DOI:** 10.1155/2013/929842

**Published:** 2013-11-19

**Authors:** Raghavan Chinnadurai, Edmund K. Waller, Jacques Galipeau, Ajay K. Nooka

**Affiliations:** ^1^Department of Hematology and Oncology, Winship Cancer Institute, Emory University, Atlanta, GA 30322, USA; ^2^Department of Pediatrics, Emory University, Atlanta, GA 30322, USA

## Abstract

The regenerative abilities and the immunosuppressive properties of mesenchymal stromal cells (MSCs) make them potentially the ideal cellular product of choice for treatment of autoimmune and other immune mediated disorders. Although the usefulness of MSCs for therapeutic applications is in early phases, their potential clinical use remains of great interest. Current clinical evidence of use of MSCs from both autologous and allogeneic sources to treat autoimmune disorders confers conflicting clinical benefit outcomes. These varied results may possibly be due to MSC use across wide range of autoimmune disorders with clinical heterogeneity or due to variability of the cellular product. In the light of recent genome wide association studies (GWAS), linking predisposition of autoimmune diseases to single nucleotide polymorphisms (SNPs) in the susceptible genetic loci, the clinical relevance of MSCs possessing SNPs in the critical effector molecules of immunosuppression is largely undiscussed. It is of further interest in the allogeneic setting, where SNPs in the target pathway of MSC's intervention may also modulate clinical outcome. In the present review, we have discussed the known critical SNPs predisposing to disease susceptibility in various autoimmune diseases and their significance in the immunomodulatory properties of MSCs.

## 1. Introduction

Mesenchymal stromal cells (MSCs) are under investigation in clinical trials to treat autoimmune disorders and degenerative disorders due to their immunomodulatory and regenerative properties. Various sources of MSCs have been described in the literature, but the one widely studied source is the bone marrow derived MSCs. MSCs in bone marrow represent 1 in 100,000 nucleated cells, but they play a significant role in regulating the niche for hematopoietic stem cells and immune homeostasis and hypothetically can differentiate into cartilage, bone, and adipocytes [1]. Major limitation for the use of MSCs in clinical trials is their low frequency in the bone marrow aspirates. This specific challenge has been addressed by MSC expansion into large quantities by virtue of their *in vitro* mitogenic properties under standard cell culture conditions [2]. This rapid expansion favors robust translational inquiry to utilize these cells in cellular therapy. To minimize ambiguity, the International Society for Cellular Therapy (ISCT) proposed minimal criteria to define human MSCs as expressing CD105, CD73, CD90 and lack of CD45, CD34, CD14 or CD11b, CD79*α* or CD19, and HLA-DR surface molecules [3].

The therapeutic potential of transfused MSCs was well demonstrated in animal models of experimental autoimmune encephalitis, diabetes, rheumatoid arthritis, myocardial infarction, acute lung injury, retinal degeneration, acute renal failure, transplant rejection, liver fibrosis, inflammatory bowel diseases, and graft versus host diseases [4, 5]. Based on these preclinical observations, therapeutic applications of MSCs are currently being explored in more than 300 clinical trials (http://www.clinicaltrials.gov/). MSCs were used for the first time to treat grafts versus host disease (GVHD) and later to treat autoimmune disorders such as Crohn's disease, multiple sclerosis, autoimmune rheumatic diseases, and autoimmune diabetes. The regenerative and anti-inflammatory properties eased their use to treat immune mediated disorders. MSCs suppress both innate and adaptive immune system as they inhibit the activation, proliferation, and also the function of lymphocytes, monocytes, dendritic cells, and natural killer cells. Previous reviews have already addressed the immunomodulatory properties of MSCs, and it is beyond the scope of current review [5–15]. 

The fundamental pathogenesis of autoimmune disorders lies on the loss of immune tolerance to self-antigens. In patients with autoimmune diseases, genetic changes in the genome of an individual affect the role of essential immunological pathways, leading to the breakdown of immune tolerance. The consequence of this effect is the inability of immune system to distinguish self- versus non-self-antigens. Genome wide association studies (GWAS) advanced the understanding of autoimmune diseases by identifying common single nucleotide polymorphisms (SNPs) and linking them to the cause of the diseases [16]. SNPs alter the phenotype and functionality of proteins in the immune system thereby affecting its function leading to disease. It is of essential interest to question the immunosuppressive properties of MSCs derived from the individuals bearing the disease causing SNPs. 

Current clinical trials utilize MSCs obtained from autologous or allogeneic origin. In the autologous setting, MSCs acquired from the bone marrow of patients with autoimmune diseases are used in the suppressor therapy. While MSCs possess multiple immunoregulatory molecules to exert suppression, the question that remains unanswered is whether the SNPs in the immunomodulatory genes of MSCs affect the clinical outcome following MSC therapy. In the allogeneic setting, bone marrow-derived MSCs are expanded and banked from the universal healthy donor and subsequently administered to the patients. Since MSCs are derived from healthy donors, it is possible that these cellular products may not possess the genetic changes associated with the disease. However, MSCs specifically act on certain immune target pathways systemically or to the inflamed site and thereby execute immunosuppressive and regenerative functions. By considering the SNPs in these targets of MSC intervention, it raises the question if SNPs in the recipient's immune pathway affect the clinical outcome. In the present review, we analyzed the common SNPs identified in the autoimmune diseases that are under investigation for MSC therapy and their significance in the mechanism of MSCs immunosuppressive effect and clinical outcome. 

## 2. Multiple Sclerosis

Multiple sclerosis (MS) is an autoimmune disorder of the central nervous system where myelin and oligodendrocytes are targeted by cell mediated and humoral immunity [17]. The beneficial effects of MSC therapy have been well described in autoimmune encephalomyelitis (EAE) mouse model that provided a basis for further exploration in clinical trials [18–20]. Currently, close to 15 clinical trials are registered to use MSC therapy for the treatment of multiple sclerosis (http://www.clinicaltrials.gov/). MSCs have been well tolerated and were deemed safe in patients with MS in the early phase clinical trials. Mallam et al. described that MSCs derived from MS patients show expansion, differentiation, and surface marker expression similar to the MSCs from healthy individuals [21]. In contrast another study reported, although MSCs from MS patients exhibit normal growth, phenotype and immunomodulatory properties, they secrete higher levels of lipopolysaccharide-stimulated IP10 compared to MSC from healthy controls [22]. These contradictory results suggest the functional differences in the MSC populations, rooted from the changes in their genetic profile, isolated from MS patients. However, the efficacy results demonstrated the evidence of structural, functional, and physiological improvement and are suggestive of neuroprotection [23, 24]. The most relevant SNPs linked with the pathogenesis of MS are harbored in the genes *HLADRB1, IL2RA, IL7R, CLEC16A, CD226, CYP27B1, MMEL1, SH2B3, CD40, CD80, CD86, and CD58 *[25]. The relevance of *IL2RA, IL7A, CYP27B1, SH2B3, and MMEL1* for MSC therapeutic activity is subtle. MSCs do not express CD40 [26]. CD80 and CD86 do not present on MSCs, and addition of IFNg does not upregulate these costimulatory molecules. Although HLADR is absent on the MSCs, IFNg upregulates its expression [27]. Considering the IFNg dependency of MSC's suppressive activity, the current unknown factor is the differences in HLADR alleles on the immunosuppressive activity of MSCs. MSCs upregulate the adhesion molecule CD58 (lymphocyte function-associated antigen) after coculture with the T cells [28]. MSCs ability to bind to the inflamed tissues is important to execute their immunosuppressive effect [29]. The significance of SNPs on adhesion molecules on MSC's engraftment potential requires further investigation.

## 3. Autoimmune Rheumatic Diseases

MSCs are under clinical investigation for the treatment of autoimmune rheumatic diseases such as rheumatoid arthritis (RA), systemic lupus erythematosus (SLE), Sjögren's syndrome, and systemic sclerosis. The animal models of collagen-induced arthritis reported varied results. Although an earlier study demonstrated that MSCs do not have any beneficial effect on mice with collagen-induced arthritis (CIA), subsequent studies demonstrated therapeutical effect [30–32]. Few other combination approaches were suggested such as conditioning of MSCs with the drugs such as bortezomib by modulating the microenvironment and thereby enhancing the therapeutic efficiency of MSCs [33]. Results of early phase ongoing clinical trials are not available to evaluate the clinical impact of MSCs in RA. Although a study suggested that stromal cell function is defective in patients with RA, another study demonstrated the immunosuppressive functions of MSCs derived from three RA patients [34, 35]. The common SNPs identified in RA and relevant to MSC biology and immunomodulation are in the genes of CD58 (adhesion molecule), IL6ST (CD130), and chemokine (C-C motif) receptor 6 [36]. IL6ST is the signal transducer in the IL-6 receptor complex to initiate the downstream signals. MSCs secrete high levels of IL-6, and it has been demonstrated that IL-6-dependent secretion of PGE2 by MSCs inhibits local inflammation in the mouse model of arthritis [31]. In addition, autocrine effect of IL-6 has been demonstrated as this cytokine enhances the survival of MSCs after serum starvation-induced apoptosis [37]. IL-6 secreted from MSCs accelerates intestinal epithelium recovery in the animal model of total body irradiation [38]. These studies highlight the significance of IL-6 in MSC immunobiology and support the need for further studies to evaluate the breadth of the signal induction with the CD130 receptor complex. MSCs have been shown to bind to Th17 cells via CCR6 and thereby induce regulatory T cell phenotype in these cells [39]. It is necessary to further investigate the SNPs in CCR6 on T cells and the immunomodulatory properties of MSCs. 

Although MSCs from the SLE patients show immunosuppressive activity, they undergo senescence relatively faster than MSC from age matched healthy controls [40, 41]. Autologous MSC therapy in two SLE patients was safe but did not reduce the disease activity [42]. A direct link between the SLE and SNPs in genes of the inflammasomes has been demonstrated [43]. However, its relevance to MSC's immunomodulatory functions has not been studied so far to rationalize and improve future clinical trials for SLE. 

Few trials evaluating the safety and efficacy of MSCs in Sjögren's syndrome and systemic sclerosis/scleroderma are ongoing. MSCs from the patients with Sjögren's syndrome show impaired immunosuppressive activity, and allogeneic MSC treatment improves disease outcome [44]. Ice et al. reviewed several SNPs linked to Sjögren's syndrome, but the relevance of these genes to MSCs therapeutic properties may be subtle [45]. Unlike Sjögren's syndrome, MSCs from scleroderma patients preserve their immunosuppressive functions [46]. SNPs associated with scleroderma and specifically SNPs in *FAS *gene is of further interest in MSC biology [47]. FAS/FASL interaction has been described in MSC's immunomodulatory properties [48]. Altogether MSC biology from autoimmune disease patients requires further investigations with the linkage to the genetic changes in the key immunomodulatory molecules.

## 4. Crohn's Disease 

The etiology of Crohn's disease (CD), an inflammatory bowel disease, is presumed alteration of genetic factors or gut microbiota or the host immune system [49]. Despite the etiology, all these factors share the common clinical manifestation of excessive intestinal inflammation. Clinical trials using MSCs as cell based inflammatory bowel suppressive therapy for CD are promising [50–53]. GWAS scan of nonsynonymous SNPs in CD has identified a mutation in the genes of autophagy [54, 55]. Autophagy is a cellular homeostatic process in which the cell compartments are recycled under stressful conditions. Recent developments have highlighted a balancing role of autophagy in the immunity and inflammation [56]. Defects in this homeostatic autophagy process may cause the basic pathogenesis of many infectious and inflammatory diseases [56]. A majority of studies indicate that autophagy plays a major role in the CD pathogenesis [57–60]. ATG16L1 protein is an important player in the autophagy process by forming the autophagosomes. In the colitis mouse model, autophagy knockout mice (ATG16L1 deficient mouse) die after Dextran Sodium Sulfate (DSS) treatment due to the excessive production of proinflammatory cytokines IL-1 beta and IL-18 [61]. In addition, CD patients with T300A SNP in ATG16L1 gene show many abnormalities in the intestinal paneth cells, which are the producers of alpha-defensins in the intestine [62]. Peripheral blood mononuclear cells from the CD patients with T300A SNP in ATGL161 gene secrete high levels of proinflammatory IL-1 beta and IL-6 upon *in vitro* stimulation [63]. Altogether these studies demonstrate the potential linkage between phenotype and SNPs in autophagy genes in CD patients. Two important studies describe the relevance of autophagy in MSC's biological properties. The first study suggested that MSCs utilize autophagic mechanism to provide tumor stromal support [64]. The second study demonstrated the role of autophagy in MSC mediated hepatic regeneration in the animal model of liver diseases [65]. The role of autophagy in the MSC's immunomodulatory properties and the significance of these SNPs in MSC's biology is currently unknown. Utilization of autologous MSC treatment and the influence of SNPs in the genes of autophagy on the clinical outcome of crohn's diseases require further investigation. 

## 5. Autoimmune Diabetes

Type I diabetes results from the immune destruction of insulin producing beta cells in the pancreatic islets of Langerhans. Although transplantation of islets of Langerhans helps to maintain the insulin levels, immunosuppressive therapy is a requirement. MSCs are under clinical investigation to treat autoimmune diabetes due their immunosuppressive and angiogenic properties and the ability to regenerate beta cells [66]. Reversal of hyperglycemia with MSC therapy has been demonstrated in a number of diabetic animal studies [67–70]. Type I diabetes associated SNPs were reported in the genes such as *IFIH1 (*interferon-induced helicase), *CTLA4, IL2RA, CLEC16A (C type lectin), and PTPN2 *[71]. Of these genes,* PTPN2 is* of significant interest to the immunomodulatory properties of MSCs*. PTPN2 *regulate signaling events by dephosphorylating multiple JAK and STAT molecules, and MSCs immunosuppressive properties are highly depend on the signal induction through IFNg [72]. Thus, the role of SNPs in *PTPN2* gene on the suppressive properties of MSCs and IFNg signaling events require further investigation.

## 6. Common SNPs in the Immunoregulatory Pathways of MSC's Intervention

The unique feature of MSCs is their array of immunoregulators, which collectively mediate the immunosuppressive and regenerative functions that impact the clinical outcome. The most important T cell regulators defined by MSCs are IDO and PDL1/PDL2-PD-1 pathways.

### 6.1. Indoleamine 2,3 Dioxygenase

Indoleamine 2,3 dioxygenase (IDO) is an enzyme of tryptophan degradation pathway which converts tryptophan to kynurenine and suppresses the T cell responses [73]. IFNg upregulates IDO in MSCs thereby not only suppresses T cell proliferation but also induces the differentiation of monocytes into suppressor phenotype [74]. IDO expression by MSCs is considered as a standard readout for the functionality of the cellular product [75]. Impairment of IDO activity in the patients with autoimmune primary biliary cirrhosis has been reported, suggesting the possible role of IDO to maintain immune tolerance [76]. Arefayene et al. reported the genetic variants of IDO-1 gene with SNPs and associated altered enzyme activity, but this study does not include any disease specific SNPs in IDO [77]. A subsequent study demonstrated that SNP rs7820268 (C6202T) in the IDO gene is statistically more frequent in systemic sclerosis patients than in controls. In addition, patients bearing this SNP in IDO show impaired CD8+ T reg function [78]. This is an important functional study, which establishes a relationship of IDO SNPs with T cell responses. Future investigations are required to study the influence of SNPs in IDO on the immunomodulatory properties of MSCs.

### 6.2. PDL1/PDL2-PD-1 Pathway

#### 6.2.1. T Cell Mediated Immune Responses

T cell activation is not only controlled by major histocompatibility complex (MHC) and T cell receptor (TCR) engagement but also by the interaction with other costimulatory molecules. PDL1/PDL2-PD-1 pathway is one such pathway which regulates the T cell tolerance in various conditions [79]. This pathway is implicated in negatively regulating T cell immunity in tumor microenvironment and chronic viral infections [80]. PD-1 is the receptor on the T cells with immunoreceptor tyrosine-based inhibiting motif (ITIM). Upon its engagement with the ligands PDL1 (B7H1) or PDL2 (B7DC), it provides negative signal to the T cells [81]. PDL1-PD1 pathway is implicated in MSC's suppression of T cell proliferation upon licensing with proinflammatory cytokine IFNg [20, 82–84]. Some studies reported that there is no correlation with SNPs in PDL1 gene and autoimmune diseases in Japanese patients. However, one study suggested that A/C polymorphism at position 8923 in PDL1 gene is associated with Graves diseases [85–87]. Similarly, another study demonstrated that SNPs in the gene for PDL2 is associated with SLE in Taiwan [88]. Since SNPs in the genes of PDL1 and PDL2 are not explicitly reported, it is possible to conclude that these ligands are intact on the surface of MSCs to execute the suppressive functions in the autologous therapy. However, the SNPs in the gene of PD-1 (*PDCD1*) are widely reported and associated with the autoimmune diseases [87]. Association between SNPs in *PDCD1* and disease susceptibility to autoimmune diseases were demonstrated in SLE [89, 90], Type I diabetes [91], RA [92, 93], MS [94], and Graves disease [95]. Kroner et al. specifically showed the functional relevance of SNPs in PDCD1 polymorphism by demonstrating the deficit in PD-1 mediated inhibition of cytokine secretion in T cells from the multiple sclerosis patients [94]. These studies clearly suggest the role of dysfunctional PDL1/PDL2-PD-1 pathway in the autoimmune patients. In the allogeneic cellular therapeutic situation, although the ligands PDL1 and PDL2 on MSCs are intact, it is possible that the SNPs in PD-1 may compromise the delivery of negative signals to T cells. Hence, PDL1/PDL2 mediated therapeutic effect by allogenic or autologous MSCs may depend on the PD1 polymorphism of the recipient which could predict the treatment responsiveness. 

#### 6.2.2. Humoral Immune Responses

T helper cells and B cell interaction plays an important role in the breakdown of peripheral tolerance in the autoimmune disorders. The helper T cells that are not sensitive to self-tolerance mechanisms secrete proinflammatory cytokines, resulting in expansion of the autoreactive B cells which produce autoantibodies to cause the self damage [96]. MSCs also affect B cell differentiation into plasma cells and subsequent immunoglobulin production [97–100]. MSCs affect the plasma cell differentiation through contact independent pathway by cleaving CCL2 in a unique mechanism [101]. Additionally, there is data suggesting that MSCs suppress B cells through PDL1/PD1 pathway [102]. Another study by Liu et al. demonstrates that periodontal ligament stem cells inhibit B cell activation through PDL1/PD1 [103]. These results suggest that stem cells act on the humoral immune responses through the PDL1/PD1 pathway. PD-1 is upregulated on the B cells after stimulation with ani-IgM and PMA/ionomycin [104]. Bertsias et al. reported that homozygous PD-1.3 SNP on the SLE patients causes lower expression of PD-1 on CD19+ B cells [105]. It is possible to speculate that lower expression of PD-1 on B cells due to PD-1.3 SNP may compromise MSC's inhibitory effect in B cells in SLE patients. Further studies are required to study the role of PD-1.3 SNP on the B cell interaction with MSCs.

## 7. Conclusion

MSCs are attractive to researchers due to their wide spectrum of immunomodulatory and regenerative properties, which collectively constitute their therapeutic activities. It is arguable that even if genetic changes such as SNPs affect one pathway, compensatory pathways may balance the functional machinery of MSCs. However, in certain situations, target pathways are crucial for the maintenance of immune tolerance, and in those conditions, MSCs could be considered as supplemental therapy along with the other immune suppressive molecules. SNPs are suggested as biomarkers for disease susceptibility in certain autoimmune disorders. Further studies are warranted in the direction of utilizing these SNPs as biomarkers for prediction of treatment responsiveness to MSC therapy.

## References

[1] Pittenger MF, Mackay MA, Beck SC (1999). Multilineage potential of adult human mesenchymal stem cells. *Science*.

[2] Francois M, Galipeau J (2012). New insights on translational development of mesenchymal stromal cells for suppressor therapy. *Journal of Cell Physiology*.

[3] Dominici M, Le Blanc K, Mueller I (2006). Minimal criteria for defining multipotent mesenchymal stromal cells. the international society for cellular therapy position statement. *Cytotherapy*.

[4] Ren G, Chen X, Dong F (2012). Concise review: mesenchymal stem cells and translational medicine: emerging issues. *Stem Cells Translational Medicine*.

[5] Uccelli A, Moretta L, Pistoia V (2008). Mesenchymal stem cells in health and disease. *Nature Reviews Immunology*.

[6] Abumaree M, Al Jumah M, Pace RA, Kalionis B (2012). Immunosuppressive properties of mesenchymal stem cells. *Stem Cell Reviews*.

[7] Stagg J, Galipeau J (2013). Mechanisms of immune modulation by mesenchymal stromal cells and clinical translation. *Current Molecular Medicine*.

[8] English K (2013). Mechanisms of mesenchymal stromal cell immunomodulation. *Immunology&Cell Biology*.

[9] Dazzi F, Lopes L, Weng L (2012). Mesenchymal stromal cells: a key player in ‘innate tolerance’?. *Immunology*.

[10] Yi T, Song SU (2012). Immunomodulatory properties of mesenchymal stem cells and their therapeutic applications. *Archives of Pharmacal Research*.

[11] De Miguel MP, Fuentes-Julián S, Blázquez-Martínez A (2012). Immunosuppressive properties of mesenchymal stem cells: advances and applications. *Current Molecular Medicine*.

[12] Duffy MM, Ritter T, Ceredig R, Griffin MD (2011). Mesenchymal stem cell effects on T-cell effector pathways. *Stem Cell Research and Therapy*.

[13] Charbord P (2010). Bone marrow mesenchymal stem cells: historical overview and concepts. *Human Gene Therapy*.

[14] Yagi H, Soto-Gutierrez A, Parekkadan B (2010). Mesenchymal stem cells: mechanisms of immunomodulation and homing. *Cell Transplantation*.

[15] Le Blanc K, Mougiakakos D (2012). Multipotent mesenchymal stromal cells and the innate immune system. *Nature Reviews Immunology*.

[16] Visscher PM, Brown MA, McCarthy MI, Yang J (2012). Five years of GWAS discovery. *American Journal of Human Genetics*.

[17] Keegan BM, Noseworthy JH (2002). Multiple sclerosis. *Annual Review of Medicine*.

[18] Zappia E, Casazza S, Pedemonte E (2005). Mesenchymal stem cells ameliorate experimental autoimmune encephalomyelitis inducing T-cell anergy. *Blood*.

[19] Zhang J, Li Y, Chen J (2005). Human bone marrow stromal cell treatment improves neurological functional recovery in EAE mice. *Experimental Neurology*.

[20] Rafei M, Campeau PM, Aguilar-Mahecha A (2009). Mesenchymal stromal cells ameliorate experimental autoimmune encephalomyelitis by inhibiting CD4 Th17 T cells in a CC chemokine ligand 2-dependent manner. *Journal of Immunology*.

[21] Mallam E, Kemp K, Wilkins A, Rice C, Scolding N (2010). Characterization of *in vitro* expanded bone marrow-derived mesenchymal stem cells from patients with multiple sclerosis. *Multiple Sclerosis*.

[22] Mazzanti B, Aldinucci A, Biagioli T (2008). Differences in mesenchymal stem cell cytokine profiles between MS patients and healthy donors: implication for assessment of disease activity and treatment. *Journal of Neuroimmunology*.

[23] Connick P, Kolappan M, Crawley C (2012). Autologous mesenchymal stem cells for the treatment of secondary progressive multiple sclerosis: an open-label phase 2a proof-of-concept study. *The Lancet Neurology*.

[24] Karussis D, Karageorgiou C, Vaknin-Dembinsky A (2010). Safety and immunological effects of mesenchymal stem cell transplantation in patients with multiple sclerosis and amyotrophic lateral sclerosis. *Archives of Neurology*.

[25] Pravica V, Popadic D, Savic E, Markovic M, Drulovic J, Mostarica-Stojkovic M (2012). Single nucleotide polymorphisms in multiple sclerosis: disease susceptibility and treatment response biomarkers. *Immunologic Research*.

[26] Ryan JM, Barry FP, Murphy JM, Mahon BP (2005). Mesenchymal stem cells avoid allogeneic rejection. *Journal of Inflammation*.

[27] Romieu-Mourez R, François M, Boivin M-N, Stagg J, Galipeau J (2007). Regulation of MHC class II expression and antigen processing in murine and human mesenchymal stromal cells by IFN-gamma, TGF-beta, and cell density. *Journal of Immunology*.

[28] Najar M, Raicevic G, Id Boufker H (2010). Modulated expression of adhesion molecules and galectin-1: role during mesenchymal stromal cell immunoregulatory functions. *Experimental Hematology*.

[29] Kang SK, Shin IS, Ko MS, Jo JY, Ra JC (2012). Journey of mesenchymal stem cells for homing: strategies to enhance efficacy and safety of stem cell therapy. *Stem Cells International*.

[30] Djouad F, Fritz V, Apparailly F (2005). Reversal of the immunosuppressive properties of mesenchymal stem cells by tumor necrosis factor alpha in collagen-induced arthritis. *Arthritis and Rheumatism*.

[31] Bouffi C, Bony C, Courties G, Jorgensen C, Noël D (2010). IL-6-dependent PGE2 secretion by mesenchymal stem cells inhibits local inflammation in experimental arthritis. *PLoS One*.

[32] Augello A, Tasso R, Negrini SM, Cancedda R, Pennesi G (2007). Cell therapy using allogeneic bone marrow mesenchymal stem cells prevents tissue damage in collagen-induced arthritis. *Arthritis and Rheumatism*.

[33] Papadopoulou A, Yiangou M, Athanasiou E (2012). Mesenchymal stem cells are conditionally therapeutic in preclinical models of rheumatoid arthritis. *Annals of the Rheumatic Diseases*.

[34] Papadaki HA, Kritikos HD, Cemetzi C (2002). Bone marrow progenitor cell reserve and function and stromal cell function are defective in rheumatoid arthritis: evidence for a tumor necrosis factor alpha-mediated effect. *Blood*.

[35] Bocelli-Tyndall C, Bracci L, Spagnoli G (2007). Bone marrow mesenchymal stromal cells (BM-MSCs) from healthy donors and auto-immune disease patients reduce the proliferation of autologous- and allogeneic-stimulated lymphocytes *in vitro*. *Rheumatology*.

[36] Ruyssen-Witrand A, Constantin A, Cambon-Thomsen A, Thomsen M (2012). New insights into the genetics of immune responses in rheumatoid arthritis. *Tissue Antigens*.

[37] Pricola KL, Kuhn NZ, Haleem-Smith H, Song Y, Tuan RS (2009). Interleukin-6 maintains bone marrow-derived mesenchymal stem cell stemness by an ERK1/2-dependent mechanism. *Journal of Cellular Biochemistry*.

[38] Francois M, Birman E, Forner KA, Gaboury L, Galipeau J (2012). Adoptive transfer of mesenchymal stromal cells accelerates intestinal epithelium recovery of irradiated mice in an interleukin-6-dependent manner. *Cytotherapy*.

[39] Ghannam S, Pène J, Torcy-Moquet G, Jorgensen C, Yssel H (2010). Mesenchymal stem cells inhibit human Th17 cell differentiation and function and induce a T regulatory cell phenotype. *Journal of Immunology*.

[40] Sun LY, Zhang HY, Feng XB, Hou YY, Lu LW, Fan LM (2007). Abnormality of bone marrow-derived mesenchymal stem cells in patients with systemic lupus erythematosus. *Lupus*.

[41] Nie Y, Lau CS, Lie AKW, Chan GCF, Mok MY (2010). Defective phenotype of mesenchymal stem cells in patients with systemic lupus erythematosus. *Lupus*.

[42] Carrion F, Nova E, Ruiz C (2010). Autologous mesenchymal stem cell treatment increased T regulatory cells with no effect on disease activity in two systemic erythematosus patients. *Lupus*.

[43] Pontillo A, Girardelli M, Kamada AJ (2012). Polimorphisms in inflammasome genes are involved in the predisposition to systemic lupus erythematosus. *Autoimmunity*.

[44] Xu J, Wang D, Liu D (2012). Allogeneic mesenchymal stem cell treatment alleviates experimental and clinical Sjogren syndrome. *Blood*.

[45] Ice JA, Li H, Adrianto I (2012). Genetics of Sjogren’s syndrome in the genome-wide association era. *Journal of Autoimmunity*.

[46] Cipriani P, Di Benedetto P, Liakouli V (2013). Mesenchymal stem cells (MSCs) from scleroderma patients (SSc) preserve their immunomodulatory properties although senescent and normally induce T regulatory cells (Tregs) with a functional phenotype: implications for cellular-based therapy. *Clinical&Experimental Immunology*.

[47] Romano E, Manetti M, Guiducci S, Ceccarelli C, Allanore Y, Matucci-Cerinic M (2011). The genetics of systemic sclerosis: an update. *Clinical and Experimental Rheumatology*.

[48] Akiyama K, Chen C, Wang D (2012). Mesenchymal-stem-cell-induced immunoregulation involves FAS-ligand-/FAS-mediated T cell apoptosis. *Cell Stem Cell*.

[49] Baumgart DC, Sandborn WJ (2012). Crohn’s disease. *Lancet*.

[50] Copland IB, Galipeau J (2011). Death and inflammation following somatic cell transplantation. *Seminars in Immunopathology*.

[51] Duijvestein M, Vos ACW, Roelofs H (2010). Autologous bone marrow-derived mesenchymal stromal cell treatment for refractory luminal Crohn’s disease: results of a phase I study. *Gut*.

[52] Ciccocioppo R, Bernardo ME, Sgarella A (2011). Autologous bone marrow-derived mesenchymal stromal cells in the treatment of fistulising crohn’s disease. *Gut*.

[53] Panés J, Ords I, Ricart E (2010). Stem cell treatment for crohns disease. *Expert Review of Clinical Immunology*.

[54] Peterson N, Guthery S, Denson L (2008). Genetic variants in the autophagy pathway contribute to paediatric crohn’s disease. *Gut*.

[55] Hampe J, Franke A, Rosenstiel P (2007). A genome-wide association scan of nonsynonymous SNPs identifies a susceptibility variant for crohn disease in ATG16L1. *Nature Genetics*.

[56] Levine B, Mizushima N, Virgin HW (2011). Autophagy in immunity and inflammation. *Nature*.

[57] Lapaquette P, Brest P, Hofman P, Darfeuille-Michaud A (2012). Etiology of crohn’s disease: many roads lead to autophagy. *Journal of Molecular Medicine*.

[58] Stappenbeck TS, Rioux JD, Mizoguchi A (2011). Crohn disease: a current perspective on genetics, autophagy and immunity. *Autophagy*.

[59] Xavier RJ, Huett A, Rioux JD (2008). Autophagy as an important process in gut homeostasis and crohn’s disease pathogenesis. *Gut*.

[60] Deretic V (2009). Links between autophagy, innate immunity, inflammation and crohn’s disease. *Digestive Diseases*.

[61] Saitoh T, Fujita N, Jang MH (2008). Loss of the autophagy protein Atg16L1 enhances endotoxin-induced IL-1beta production. *Nature*.

[62] Cadwell K, Liu JY, Brown SL (2008). A key role for autophagy and the autophagy gene Atg16l1 in mouse and human intestinal paneth cells. *Nature*.

[63] Plantinga TS, Crisan TO, Oosting M (2011). Crohn’s disease-associated ATG16L1 polymorphism modulates pro-inflammatory cytokine responses selectively upon activation of NOD2. *Gut*.

[64] Sanchez CG, Penfornis P, Oskowitz AZ (2011). Activation of autophagy in mesenchymal stem cells provides tumor stromal support. *Carcinogenesis*.

[65] Jung J, Choi JH, Lee Y (2013). Human placenta-derived mesenchymal promote hepatic regeneration in CCl_4_ -injured rat liver model via increased autophagic mechanism. *Stem Cells*.

[66] Dominguez-Bendala J, Lanzoni G, Inverardi L, Ricordi C (2012). Concise review: mesenchymal stem cells for diabetes. *Stem Cells Translational Medicine*.

[67] Ezquer FE, Ezquer ME, Parrau DB, Carpio D, Yañez AJ, Conget PA (2008). Systemic administration of multipotent mesenchymal stromal cells reverts hyperglycemia and prevents nephropathy in type 1 diabetic mice. *Biology of Blood and Marrow Transplantation*.

[68] Madec AM, Mallone R, Afonso G (2009). Mesenchymal stem cells protect NOD mice from diabetes by inducing regulatory T cells. *Diabetologia*.

[69] Fiorina P, Jurewicz M, Augello A (2009). Immunomodulatory function of bone marrow-derived mesenchymal stem cells in experimental autoimmune type 1 diabetes. *Journal of Immunology*.

[70] Jurewicz M, Yang S, Augello A (2010). Congenic mesenchymal stem cell therapy reverses hyperglycemia in experimental type 1 diabetes. *Diabetes*.

[71] Concannon P, Rich SS, Nepom GT (2009). Genetics of type 1A diabetes. *The New England Journal of Medicine*.

[72] Krampera M, Cosmi L, Angeli R (2006). Role for interferon-gamma in the immunomodulatory activity of human bone marrow mesenchymal stem cells. *Stem Cells*.

[73] Munn DH, Mellor AL (2007). Indoleamine 2,3-dioxygenase and tumor-induced tolerance. *Journal of Clinical Investigation*.

[74] François M, Romieu-Mourez R, Li M, Galipeau J (2012). Human MSC suppression correlates with cytokine induction of indoleamine 2,3-dioxygenase and bystander M2 macrophage differentiation. *Molecular Therapy*.

[75] Krampera M, Galipeau J, Shi Y, Tarte K, Sensebe L (2013). Immunological characterization of multipotent mesenchymal stromal cells-The International Society for Cellular Therapy (ISCT) working proposal. *Cytotherapy*.

[76] Oertelt-Prigione S, Mao TK, Selmi C (2008). Impaired indoleamine 2,3-dioxygenase production contributes to the development of autoimmunity in primary biliary cirrhosis. *Autoimmunity*.

[77] Arefayene M, Philips S, Cao D (2009). Identification of genetic variants in the human indoleamine 2,3-dioxygenase (IDO1) gene, which have altered enzyme activity. *Pharmacogenetics and Genomics*.

[78] Tardito S, Negrini S, Conteduca G (2013). Indoleamine 2,3 dioxygenase gene polymorphisms correlate with CD8^+^ Treg impairment in systemic sclerosis. *Human Immunology*.

[79] Francisco LM, Sage PT, Sharpe AH (2010). The PD-1 pathway in tolerance and autoimmunity. *Immunological Reviews*.

[80] Sharpe AH, Wherry EJ, Ahmed R, Freeman GJ (2007). The function of programmed cell death 1 and its ligands in regulating autoimmunity and infection. *Nature Immunology*.

[81] Greenwald RJ, Freeman GJ, Sharpe AH (2005). The B7 family revisited. *Annual Review of Immunology*.

[82] Najar M, Raicevic G, Kazan HF (2012). Immune-related antigens, surface molecules and regulatory factors in human-derived mesenchymal stromal cells: the expression and impact of inflammatory priming. *Stem Cell Reviews*.

[83] Sheng H, Wang Y, Jin Y (2008). A critical role of IFNgamma in priming MSC-mediated suppression of T cell proliferation through up-regulation of B7-H1. *Cell Research*.

[84] Tipnis S, Viswanathan C, Majumdar AS (2010). Immunosuppressive properties of human umbilical cord-derived mesenchymal stem cells: role of B7-H1 and IDO. *Immunology and Cell Biology*.

[85] Ni R, Ihara K, Miyako K (2007). PD-1 gene haplotype is associated with the development of type 1 diabetes mellitus in Japanese children. *Human Genetics*.

[86] Hayashi M, Kouki T, Takasu N, Sunagawa S, Komiya I (2008). Association of an A/C single nucleotide polymorphism in programmed cell death-ligand 1 gene with graves’ disease in Japanese patients. *European Journal of Endocrinology*.

[87] Gianchecchi E, Delfino DV, Fierabracci A (2013). Recent insights into the role of the PD-1/PD-L1 pathway in immunological tolerance and autoimmunity. *Autoimmunity Reviews*.

[88] Wang SC, Lin CH, Ou TT (2007). Ligands for programmed cell death 1 gene in patients with systemic lupus erythematosus. *The Journal of Rheumatology*.

[89] Prokunina L, Castillejo-López C, Öberg F (2002). A regulatory polymorphism in PDCD1 is associated with susceptibility to systemic lupus erythematosus in humans. *Nature Genetics*.

[90] Nielsen C, Laustrup H, Voss A, Junker P, Husby S, Lillevang ST (2004). A putative regulatory polymorphism in PD-1 is associated with nephropathy in a population-based cohort of systemic lupus erythematosus patients. *Lupus*.

[91] Nielsen C, Hansen D, Husby S, Jacobsen BB, Lillevang ST (2003). Association of a putative regulatory polymorphism in the PD-1 gene with susceptibility to type 1 diabetes. *Tissue Antigens*.

[92] Lin SC, Yen JH, Tsai JJ (2004). Association of a programmed death 1 gene polymorphism with the development of rheumatoid arthritis, but not systemic lupus erythematosus. *Arthritis and Rheumatism*.

[93] Prokunina L, Padyukov L, Bennet A (2004). Association of the PD-1.3A allele of the PDCD1 gene in patients with rheumatoid arthritis negative for rheumatoid factor and the shared epitope. *Arthritis and Rheumatism*.

[94] Kroner A, Mehling M, Hemmer B (2005). A PD-1 polymorphism is associated with disease progression in multiple sclerosis. *Annals of Neurology*.

[95] Newby PR, Roberts-Davies EL, Brand OJ (2007). Tag SNP screening of the PDCD1 gene for association with graves’ disease. *Clinical Endocrinology*.

[96] Nagafuchi S (2010). The role of B cells in regulating the magnitude of immune response. *Microbiology and Immunology*.

[97] Corcione A, Benvenuto F, Ferretti E (2006). Human mesenchymal stem cells modulate B-cell functions. *Blood*.

[98] Tabera S, Pérez-Simón JA, Díez-Campelo M (2008). The effect of mesenchymal stem cells on the viability, proliferation and differentiation of B-lymphocytes. *Haematologica*.

[99] Asari S, Itakura S, Ferreri K (2009). Mesenchymal stem cells suppress B-cell terminal differentiation. *Experimental Hematology*.

[100] Franquesa M, Hoogduijin MJ, Bestard O, Grinyó JM (2012). Immunomodulatory effect of mesenchymal stem cells on B cells. *Frontiers Immunology*.

[101] Rafei M, Hsieh J, Fortier S (2008). Mesenchymal stromal cell derived CCL2 suppresses plasma cell immunoglobulin production via STAT3 inactivation and PAX5 induction. *Blood*.

[102] Schena F, Gambini C, Gregorio A (2010). Interferon-*γ*-dependent inhibition of B cell activation by bone marrow-derived mesenchymal stem cells in a murine model of systemic lupus erythematosus. *Arthritis and Rheumatism*.

[103] Liu O, Xu J, Gang D (2013). Periodontal ligament stem cells regulate B lymphocyte function via programmed cell death protein 1. *Stem Cells*.

[104] Agata Y, Kawasaki A, Nishimura H (1996). Expression of the PD-1 antigen on the surface of stimulated mouse T and B lymphocytes. *International Immunology*.

[105] Bertsias GK, Nakou M, Choulaki C (2009). Genetic, immunologic, and immunohistochemical analysis of the programmed death 1/programmed death ligand 1 pathway in human systemic lupus erythematosus. *Arthritis and Rheumatism*.

